# Epigenetic Effect of Environmental Factors on Autism Spectrum Disorders

**DOI:** 10.3390/ijerph13050504

**Published:** 2016-05-14

**Authors:** Takeo Kubota, Kazuki Mochizuki

**Affiliations:** 1 Department of Epigenetic Medicine, Faculty of Medicine, University of Yamanashi, 1110 Shimokato, Chuo, Yamanashi 409-3898, Japan; 2 Department of Local Produce and Food Sciences, Faculty of Life and Environmental Sciences, University of Yamanashi, 4-4-37 Takeda, Kofu-City, Yamanashi 400-8510, Japan; mochizukik@yamanashi.ac.jp

**Keywords:** autism spectrum disorder, epigenetics, endocrine-disrupting chemicals, early life exposure, mental stress, maternal diet, neurotransmitters, immune dysregulation

## Abstract

Both environmental factors and genetic factors are involved in the pathogenesis of autism spectrum disorders (ASDs). Epigenetics, an essential mechanism for gene regulation based on chemical modifications of DNA and histone proteins, is also involved in congenital ASDs. It was recently demonstrated that environmental factors, such as endocrine disrupting chemicals and mental stress in early life, can change epigenetic status and gene expression, and can cause ASDs. Moreover, environmentally induced epigenetic changes are not erased during gametogenesis and are transmitted to subsequent generations, leading to changes in behavior phenotypes. However, epigenetics has a reversible nature since it is based on the addition or removal of chemical residues, and thus the original epigenetic status may be restored. Indeed, several antidepressants and anticonvulsants used for mental disorders including ASDs restore the epigenetic state and gene expression. Therefore, further epigenetic understanding of ASDs is important for the development of new drugs that take advantages of epigenetic reversibility.

## 1. Introduction

Autism spectrum disorders (ASDs) are complex, pervasive neurodevelopmental disorders that are characterized by dysfunctions in social interactions and communications and restricted/fixated interests or repetitive behavior that manifest in early childhood [1]. ASDs include classical autism, Asperger syndrome, and pervasive developmental disorder-not otherwise Specified [2,3].

A number of environmental factors are known to be involved in the pathogenesis of ASDs, including nutritional factors and hormones [4]. Furthermore, inappropriate child rearing, such as child abuse and malnutrition by parents with psychiatric problems, can be associated with ASDs [5,6,7,8]. Viral infections with rubella and cytomegalovirus and associated immunological reactions via activation of microglia are also thought to be involved in ASDs, which has been demonstrated by pathological studies of post-mortem brains and neuroimaging studies of ASD patients [9,10,11,12,13,14], although some epidemiological studies conducted in Denmark and Taiwan did not support the hypothesis that pre- and postnatal infection and immunological reaction are involved in ASD cases with regard to herpes and influenza viral infection and Kawasaki Disease (a disorder potentially associated with corona virus) [15,16,17]. In addition, endocrine-disrupting chemicals (EDCs) are thought to be involved in the development of ASDs, including tobacco, air pollutants, solvents, metals, pesticides, and organic EDCs such as flame retardants, non-stick chemicals, phthalates, and bisphenol A (BPA) [18].

Conversely, a number of genetic factors have been identified as causes of ASDs. Mutations in genes encoding neurotransmitters such as synapsin, dopamine transporter, and neuroligin, and synapse-associated proteins such as scaffold proteins including shank and lin7B, have been identified in ASD patients [19,20,21,22,23,24]. Unexpectedly, mutations have also been identified in chromatin-remodeling factors such as histone modifying enzymes and chromodomain helicases in congenital ASDs (e.g., Kleefstra syndrome) [25,26,27]. These findings suggest that ASDs can be recognized as a “synaptic and chromatin-remodeling disorders” [25,28].

Chromatin is a genetic unit that consists of DNA and histone proteins, which are modified by enzymes for DNA methylation, histone acetylation and methylation and by chromatin-binding polycomb proteins. A recent three-dimensional resolution imaging technology provided a precise chromatin organization with epigenetic modifications [29]. Furthermore, autism susceptibility candidate 2, a nuclear protein involved in cortical neuronal migration and neuritogenesis in the developing brain [30] and whose mutations cause ASDs [31,32], forms a complex with polycomb repressive complex 1 to purge its repressive function and activates expression of neurodevelopmental genes involved in axon guidance in the developing forebrain, such as neruocan [33,34]. These results suggest that close interaction between neuronal molecules and epigenetic molecules is important for normal brain development and failure of this interaction is potentially associated with ASDs.

In this review, we introduce congenital epigenetic disorders with ASD-like phenotypes and environmental factors that affect epigenetic regulation of neuronal genes, and discuss transgenerational epigenetic inheritance and therapeutic strategies for ASDs taking advantage of use of the epigenetic reversibility.

## 2. Congenital Epigenetic Diseases

Rett syndrome (RTT) is a representative ASD characterized by repetitive and stereotypic hand movements, seizures, gait ataxia and autism [35] and is caused by mutations in the gene that encode methyl-CpG-binding protein 2 (MeCP2), which is associated with chromatin remodeling [36]. Since RTT is an X-linked dominant disorder, male patients are embryonic lethal and thus all patients are female.

MeCP2 interacts with the Sin3A/HDAC complex [37,38,39,40], and binds to methylated CpG in DNA to suppress a number of genes associated with synaptic function (e.g., *BDNF*, *DLX5*, *ID*, *CRH*, *IGFBP3*, *CDKL1*, *PCDHB1* and *PCDH7*, *LIN7A*) in neurons and other types of brain cells [41,42,43,44,45,46,47], thereby controlling excitatory synaptic strength by regulating the number of glutamatergic synapses [48].

**Figure 1:**
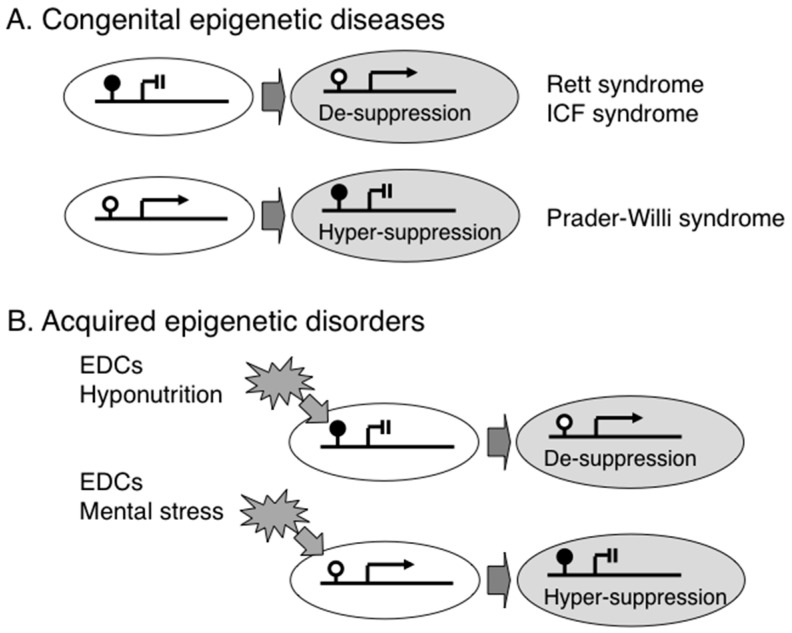
Epigenetic gene regulation in congenital epigenetic diseases and acquired epigenetic disorders. (**A**) Deficiency of DNA-binding protein or DNA methylation causes Rett syndrome (an ASD) or ICF syndrome (an immunodeficiency disease), respectively. Congenital aberrant DNA methylation due to genomic imprinting error causes Prader-Willi syndrome; and (**B**) Various environmental factors such as endocrine disrupting chemicals (EDCs), hyponutrition, and mental stress are known to alter epigenetic status, resulting in aberrant gene expression.

Induced pluripotent stem cells (iPSCs) can be used to determine how a disease develops in patients, especially inaccessible brain cells. Using iPSC technology, it is possible to generate neural cells from patients’ peripheral tissue such as skin fibroblasts. Several studies have shown that RTT iPSC-derived neurons exhibit maturation and electro-physiological defects reminiscent of those seen in RTT patients and mouse models [49,50,51], and we have shown that astrocyte-specific genes (e.g., *GFAP*) are aberrantly expressed in neural cells generated from iPSC lines that lack MeCP2 expression, which leads to the de-suppression of astrocyte-specific genes (Figure 1A) [52].

Interestingly, not only functional deficiency of MeCP2 protein (*i.e.*, due to mutations of *MECP2*) but also increased dosage of MeCP2 protein (*i.e.*, due to duplication of *MECP2*) results in severe mental retardation in males [53] and cognitive impairment with learning difficulties and speech delay in females [54]. The increased dosage effect of *Mecp2* on neurological function has been confirmed in a model mouse that exhibits motor coordination deficits, heightened anxiety, and impairments of learning and memory [55], and in a monkey model that exhibits a higher frequency of repetitive circular locomotion, increased stress responses, less interaction with wild-type monkeys, reduced interaction time with other transgenic monkeys, and stereotypic cognitive behaviors [56,57]. These findings indicate that the expression of *MECP2* within a normal range is essential for normal brain development.

ICF syndrome is a congenital disorder named after three major features, such as Immunodeficiency, Centromere instability, and Facial anomalies [58]. Although the cause is different between RTT and ICF syndromes, the consequence is similar; both lead to the de-suppression of target genes by the failure of DNA methylation-dependent gene regulation (Figure 1A). ICF syndrome is diagnosed by specific chromosome findings with breakage of the pericentric heterochromatic regions of chromosomes 1, 9 and 16, which are normally hypermethylated but are hypomethylated due to deficiency of DNMT3B in ICF [59]. The patients show distinct low levels of immunoglobulins (e.g., IgG and IgA) and they required intravenous immunoglobulin supplementation every 2 weeks. Although a recent study has demonstrated an ICF-specific DNA hypomethylation pattern in mesenchymal stem cells differentiated from the iPSCs of ICF patients [60] and another study has shown a subset of hypomethylated genes in ICF patients [61], the precise molecular mechanism for the immune dysregulation, which is the main clinical feature in ICF, is still largely unknown. It may be necessary to analyze purified B lymphocytes from ICF patients in order to identify hypomethylated DNMT3B-driven immunological genes. Interestingly, mutations in a gene encoding another DNA methyltransferase, *DNMT3A*, cause intellectual disability with overgrowth [62], suggesting that DNA methyltransferases are essential for normal brain and immunological development.

Prader-Willi syndrome (PWS) is a hallmark epigenetic disease; the causative epigenetic abnormality was identified more than 20 years ago. Approximately 70% patients have a chromosomal deletion at 15q11-q13, and the remaining patients have genomic imprinting errors. In PWS patients with maternal uniparental disomy, both paternal and maternal alleles of genes within the 15q11-q13 region are hypermethylated and thus expression from both alleles is suppressed (Figure 1A) [63,64,65]. The clinical features of PWS includes neurocognitive deficits, excessive daytime sleepiness, muscle hypotonia, short stature, small hands and feet, hypergonadism, hyperphagia starting from infancy, and subsequent obesity and type 2 diabetes [66].

Angelman syndrome is characterized by severe intellectual disability, intractable epilepsy, puppet-like ataxic movement, and paroxysms of laughter. The critical region is the same as PWS (*i.e.*, 15q11-q13), but parental-of-origin is different; either maternal deletion or paternal uniparental disomy causes Angelman syndrome, because the causative gene, ubiquitin protein ligase E3A (*UBE3A*), is maternally expressed [67]. Interestingly, the increased copy number (*i.e.*, duplication or triplication) of the maternal 15q11-q13 region that leads to *UBE3A* overexpression causes an ASD [68]. These findings indicate that the expression of *UBE3A* within a normal range is essential for normal brain development.

Epigenomic studies were conducted within the regions of various neuronal genes and ASD-specific differential DNA methylation was revealed. For example, increased DNA methylation at the promoter regions subsequent reduced expression were observed within the genes of oxytocin receptor (*OCTR*), Engrailed-2 (*EN2*) and Reelin (*RELN*) in the postmortem brain tissues from ASD patients [69,70,71]. Increased hydroxymethylation and subsequent increased binding of MeCP2 associated with gene silencing were identified within the promoter region of glutamate decarboxylase 1 (*GAD1*) in the postmortem brain tissues from ASD patients [71].

Recent genome-wide DNA methylation studies using array-based Infinium BeadChip identified ASD-associated differential DNA methylation at *ZFP57* associated with folate metabolism, which is a potential contributor to ASD risk, in the postmortem brain tissues [72,73,74], and at brain-derived neurotrophic factor (*BDNF*) in the peripheral blood tissues from ASD patients [75]. Findings through these studies potentially generate robust epigenetic biomarkers for risk, diagnosis and prognosis of ASD, which may also be used to monitor response to early interventions [76,77].

## 3. Acquired Epigenetic Disorders

As mentioned above, not only genetic factors (*i.e.*, DNA mutations) but also environmental factors are involved in ASDs, and a combination of heritability (G: genetic factor such as single nucleotide polymorphism) and experience (E: environmental factor)—that is, “G, X, E”—has been the main concept for understanding common diseases, including ASDs. Recently, new G X E model has been proposed in which E dynamically changes G and causes DNA and histone chemical modifications (*i.e.*, epigenetics), but not DNA sequence changes [78].

EDCs are compounds released from chemical, agricultural, pharmaceutical, and consumer product industries that have estrogenic activity or interfere with endogenous sex hormones. Of the many EDCs, BPA is associated with reproductive toxicity, altered growth, and immune dysregulation, and alters DNA methylation in fetal mouse brains [79]. Moreover, perinatal BPA exposure via maternal diet decreases global DNA methylation in bone marrow-derived mast cells of the offspring during adulthood [80], and it alters DNA methylation of *Stat3* dose in a dose dependent manner in mouse liver [81]. High dose exposure of polybrominated diphenyl ethers (flame retardants) decreases DNA methylation at the promoter of *TNFα*, a proinflammatory gene, and increase TNFα protein expression in human cord blood [82]. Furthermore, the altered DNA methylation patterns in *AHRR*, *MYO1G*, *CYP1A1*, and *CNTNAP2* caused by maternal tobacco smoking detected in cord blood was confirmed in the peripheral blood of their children at 17 years of age [83], suggesting that altered DNA methylation in the early development period can persist for a long period and it may be useful as a long-lasting signature of maternal stress or history of the offspring.

Nutrition also influences programming of an offspring’s epigenome, which includes folic acid and vitamins B2, B6 and B12 that are essential for one-carbon metabolism and are involved in DNA methylation (Figure 1B). Moreover, a calorie- or protein-restricted maternal diet decreases DNA methylation and induces the over-expression of energy storage-associated genes (e.g., *PPARγ*) in fetal liver to generate a “thrifty phenotype,” which promotes survival under conditions of poor nutrition before and after birth [84,85]. Once an individual is born with a thrifty phenotype in modern society with an abundance of food, the nutritional mismatch between prenatal and postnatal conditions induces metabolic and mental disorders [86,87,88,89], the concept of which is referred to as “Developmental Origins of Heath and Disease (DOHaD)” [90].

Several lines of evidence suggest that not only materials (e.g., chemicals and nutrients) but also mental stresses can alter an offspring’s epigenome (Figure 1B). For example, exposure of short-term postnatal stress by separating offspring from the mother induced hypermethylation within the promoter region of the glucocorticoid Receptor (*NR3C1*) gene, which encodes a hormone associated with resilience, in the hippocampal region of the offspring, leading to abnormal behavior in rats [91]. Furthermore, exposure to prenatal maternal stress also predicts a wide variety of behavioral and physical outcomes in the offspring. A recent study of women who were pregnant during a disaster (the ice storm in Quebec in 1998) revealed that DNA methylation profiles were altered in genes related to immune function in the peripheral blood of their offspring [92]. Similarly, it has been demonstrated that maternal stress during pregnancy alters DNA methylation of the imprinted genes *IGF2* and *GNASXL* in cord blood [93], and that maternal stress also alters DNA methylation in *NR3C1* and *BDNF* in buccal mucosa DNA samples obtained from 2 month-old infants born to mothers with depressive symptoms during pregnancy [94,95].

## 4. Transgenerational Epigenetic Inheritance

Environmental factors that alter a phenotype not only affect the exposed individual but also subsequent progeny for successive generations. In other words, ancestral experiences could influence subsequent generations, the concept of which is termed “transgenerational inheritance.” Furthermore, environmental factors such as EDCs and nutrition do not promote genetic mutations but instead promote epigenetic changes; the permanent programming of an altered epigenome in the germline can allow for the transmission of transgenerational epigenetic phenotypes [96]. The evidence supports the theory of Lamarckian inheritance in which an organism can pass on phenotypes that it acquired during its lifetime to its offspring. More precisely, a hypothesis has emerged that environmental stress results in epigenetic changes at some loci in the genome and these can escape the epigenetic reprogramming that normally occurs between generations [97,98].

Short-term postnatal mental stress by separating offspring from their mother alters DNA methylation not only in the brain but also in the sperm of male offspring, and then the environmentally induced epigenetic and expression alterations of *Crfr2* are transmitted up to the third generation (F1 sperm and F2 brain) along with behavioral abnormalities [99]. Since this initial observation, similar findings have accumulated. For example, prenatal stress exposure induces changes in DNA methylation and miRNA expression in the placenta and brain, which leads to an increase in risk for schizophrenia, attention deficit hyperactivity disorder, ASDs, and anxiety- or depression-related disorders later in life [100]. Besides mental stress, exposure to an EDC (e.g., vinclozolin) during embryonic gonadal sex determination can alter male germ-line epigenetics, and the alteration of DNA methylation in the germ line appears to result in the transmission of transgenerational adult-onset diseases, including spermatogenic defects, prostate disease, kidney disease and cancer [101]. A recent study demonstrated that exposure to BPA in early life induces glucose intolerance and β-cell dysfunction, with hypermethylation and associated decreased expression of IGF2 in the islets of male F2 offspring; this finding suggests that BPA exposure during early life can result in generational transmission of glucose intolerance and β-cell dysfunction through the male germ line by an epigenetic mechanism [102].

However, evidence that such effects persist in the subsequent generations has been inconclusive [97,103,104]. The effects must be observed in the F3 generation to be considered transgenerational, because the *in utero* nature of the ancestral perturbation affects not only the somatic and germ cells of the developing F1 fetus, but also the germ cells of the F2 generation. In this context, a recent study demonstrated that treatment of pregnant mice with the EDC methoxychlor altered the methylation of all imprinted genes examined (*i.e.*, *H19*, *Meg3 (Gtl2*), *Mest* (*Peg1*), *Snrpn*, and *Peg3*) in the F1 offspring, but these effects disappeared gradually from F1 to F3 [105]. These finding suggests that transgenerational epigenetic inheritance is not “solid” inheritance, such as genetic (DNA sequence-based) inheritance, but “soft” inheritance [106,107].

## 5. Conclusions

In this article, we have introduced ASDs with epigenetic abnormalities caused by genetic mutations in enzymes and proteins involved or chromosomal abnormalities such as Rett and Prader-Willi syndromes (*i.e.*, congenital and syndromic ASDs) and ASDs with epigenetic abnormalities caused by environmental factors such as chemicals, nutrition, and mental stress (*i.e.*, acquired and non-syndromic ASDs). Furthermore, we introduced the concept of transgenerational epigenetic inheritance in which environmental stress-induced epigenetic changes can be transmitted to the subsequent generations by escaping from erasure during epigenetic reprogramming. However, transgenerational epigenetic inheritance is not “solid” inheritance but “soft” inheritance because epigenetics is a reversible mechanism based on the addition and removal of chemical residues on DNA and histone proteins.

Taking advantage of this epigenetic reversibility, some psychotropic drugs, such as valproic acid for epilepsy and mental disorders and imipramine for depressive disorders, can restore altered histone modifications and gene expression [108,109,110]. A recent epidemiological study demonstrated that supplementation of folic acid during pregnancy, which is an important nutrient and substrate for DNA methylation, reduced the risk of ASDs in the offspring [111]. Furthermore, studies using RTT or MeCP2-duplication mouse models demonstrated that genetic supplementation of MeCP2, bone marrow transplantation, or antisense oligonucleotides after birth successfully attenuated neurological symptoms [112,113,114]. These findings support the idea that the phenotypes of ASDs caused by epigenetic dysregulation are reversible and thus treatable. Further epigenetic understanding of ASDs will offer new concepts for therapeutic strategies.
